# Course and predictors of supportive care needs among Mexican breast cancer patients: A longitudinal study

**DOI:** 10.1002/pon.4778

**Published:** 2018-06-19

**Authors:** Adriana Pérez‐Fortis, Joke Fleer, Maya J. Schroevers, Patricia Alanís López, Juan José Sánchez Sosa, Christine Eulenburg, Adelita V. Ranchor

**Affiliations:** ^1^ University of Groningen, University Medical Center Groningen Health Psychology Section Groningen The Netherlands; ^2^ University of Groningen, University Medical Center Groningen Department of Epidemiology, Medical Statistics and Decision Making Groningen The Netherlands; ^3^ National Medical Center “La Raza”, Gynecology and Obstetrics Hospital Mexican Institute of Social Security (IMSS) Mexico City Mexico; ^4^ Faculty of Psychology National University of Mexico (UNAM) Mexico City Mexico

**Keywords:** breast cancer, Latinas, needs assessment, oncology, supportive care

## Abstract

**Objective:**

This study examined the course and predictors of supportive care needs among Mexican breast cancer patients for different cancer treatment trajectories.

**Methods:**

Data from 172 (66.4% response rate) patients were considered in this observational longitudinal study. Participants were measured after diagnosis, neoadjuvant treatment, surgery, adjuvant treatment, and the first post‐treatment follow‐up visit. Psychological, Health System and Information, Physical and Daily Living, Patient Care and Support, Sexual, and Additional care needs were measured with the Supportive Care Needs Survey (SCNS‐SF34). Linear mixed models with maximum‐likelihood estimation were computed.

**Results:**

The course of supportive care needs was similar across the different cancer treatment trajectories. Supportive care needs declined significantly from diagnosis to the first post‐treatment follow‐up visit. Health System and Information care needs were the highest needs over time. Depressive symptoms and time since diagnosis were the most consistent predictors of changes in course of supportive care needs of these patients.

**Conclusions:**

Health system and information care needs of Mexican breast cancer patients need to be addressed with priority because these needs are the least met. Furthermore, patients with high depressive symptoms at the start of the disease trajectory have greater needs for supportive care throughout the disease trajectory.

## 1 BACKGROUND

Supportive care refers to the care provided along with the medical treatment at any point during the disease trajectory, and it is focused on meeting the patients' psychological, spiritual, supportive, informational, and practical needs.^1^ In Latin America, where 7.8% of worldwide new cancer cases occur and resources allocated to health care are generally limited, the implementation of supportive care is not common practice and is often a low priority.^2^, ^3^ Identifying the priority care needs of these cancer patients is relevant to improve care provision, especially in Mexico, where breast cancer is highly prevalent and it is among the 3 leading causes of death in Mexican women.^4^, ^5^ However, there are no previous longitudinal studies addressing supportive care needs of cancer patients in Latin America. Longitudinal research among Asian and European breast cancer patients showed that supportive care needs may change during active treatment,^6^, ^7^ and in the survivorship phase.^8^ Besides, cross‐sectional research showed differences in unmet care needs between Asian and European breast cancer patients.^9^

A longitudinal study among Taiwanese breast cancer patients in early stages of the disease and undergoing active treatment showed that supportive care needs decreased from diagnosis up to 3‐month follow‐up post‐diagnosis.^6^ By contrast, another study with Chinese breast cancer patients in more advanced stages of the disease receiving chemotherapy (baseline) and who were followed up to 12‐months post‐baseline showed that most patients reported stable low needs in all the supportive care needs domains, with only a few patients showing an increase in care needs for the Psychological and Physical and Daily Living domains.^7^ Supportive care needs among French breast cancer survivors evaluated in the last week of primary treatment and 4 and 8 months later showed low decreasing Health System and Information care needs, medium stable Psychological or Physical and Daily Living care needs, low stable Patient Care and Support needs, and no need stable Sexual care needs.^8^ These findings show that supportive care needs may fluctuate in breast cancer patients under treatment or in the survivorship phase. However, interpretation of their relevance for clinical practice is complicated because measurements were scheduled on a time‐basis from diagnosis or baseline measure, without considering that key meaningful events (eg, neoadjuvant treatment, surgery, adjuvant treatment) occur in different moments for each patient during active treatment and may elicit care needs. Additionally, these studies did not distinguish between the different treatment trajectories, included only patients in an advanced stage of the disease^7^ or in the survivorship phase,^8^ which prevents us from getting a complete picture of the supportive care needs throughout different disease trajectories.

The present study assessed the course of supportive care needs among Mexican breast cancer patients with measurements scheduled after meaningful events. More precisely, we wanted to investigate whether the course of supportive care needs was different for patients depending on the treatment trajectory they followed (ie, group A: only surgery, group B: surgery plus adjuvant treatment, or group C: neoadjuvant treatment, surgery, and adjuvant treatment). Additionally, we explored potential predictors of changes in the supportive care needs of Mexican breast cancer patients, as earlier cross‐sectional and longitudinal research among cancer patients showed that sociodemographic, psychological, and medical characteristics were related to care needs.^7^, ^8^, ^10^

## 2 METHODS

### 2.1 Design

We conducted an observational longitudinal study with measurements scheduled after diagnosis, after finishing each treatment modality, and at the first post‐treatment follow‐up visit. Breast cancer patients differ in treatment trajectories depending on cancer stage and several prognostic factors.^11^ Overall, patients with advanced cancer stages and worse prognostic factors follow more intensive treatment trajectories compared with patients with earlier cancer stages or better prognostic factors. Thus, treatment trajectories are intertwined with cancer stage. Based on the treatment trajectories that the patients in our study followed, we identified 3 groups. Group A are the patients who followed surgery only, after the diagnosis. Group B are the patients who followed surgery and adjuvant treatment. Group C are the patients who followed neoadjuvant treatment, surgery, and adjuvant treatment. The assessments were conducted after diagnosis but before surgery (T1); after end of neoadjuvant treatment (T2); after surgery and before start of adjuvant treatment (T3); at the end of the adjuvant treatment (either chemotherapy, radiotherapy or the combination of both) (T4); at first post‐treatment follow‐up appointment (T5). Depending on these treatment trajectories, patients had 3 (group A), 4 (group B), or 5 measurements (group C). Some patients within each group were following hormone therapy, but this treatment was not taken into account in the study due to its long duration.

### 2.2 Participants and procedure

After approval from the research and ethics committee of the hospital (R‐2014‐3504‐40), breast cancer patients were recruited consecutively in a public hospital in Mexico City from May 2014 to July 2015. Data collection was completed in November 2016. Inclusion criteria were (1) age between 18 and 75 years, (2) first breast cancer diagnosis, confirmed by a biopsy test, and (3) comprehension of Spanish. Exclusion criteria were (1) presence of a psychiatric disorder that implied hospital admission, (2) having a cancer recurrence, (3) already had surgery, (4) participation in another study at time of inclusion, and (5) being male.

**Figure 1:**
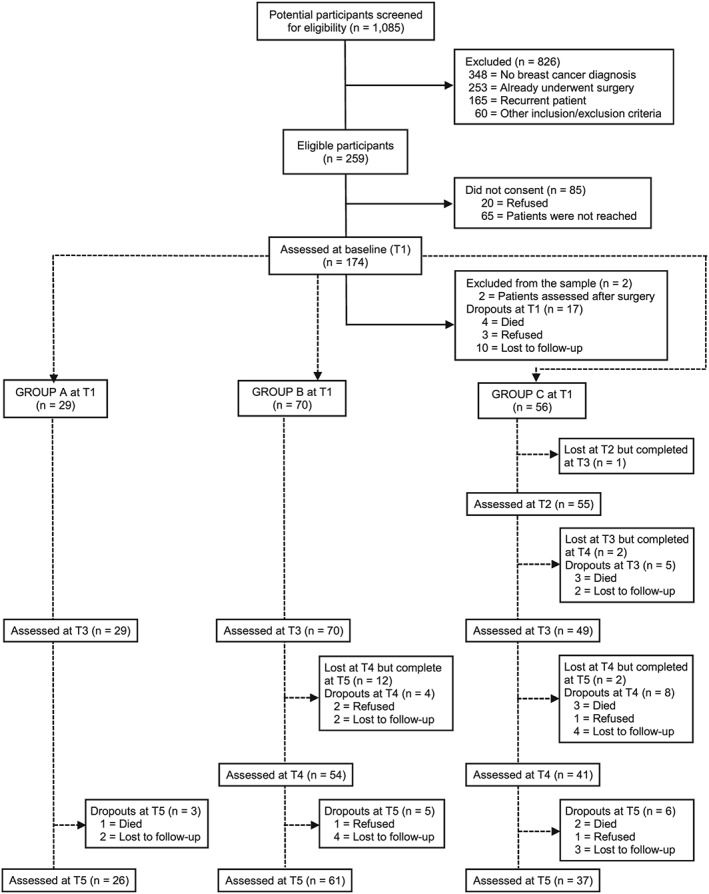
Flow chart of the patients' follow‐up

After patients provided written informed consent, they were approached and mainly face‐to‐face interviewed in the hospital and a few by telephone (T2: 1.2%, T3: 4.7%, T4: 8.1%, T5: 7.6%) by 3 graduated psychologists. The whole follow‐up lasted between 4 and 20 months, depending on the treatment trajectory of each patient. A flowchart of the patient's participation in the study is shown in Figure 1. Further details about the sample recruitment can be consulted elsewhere.^12^

### 2.3 Measurements

*Supportive care needs* were measured at each measure point with the Supportive Care Needs Survey ‐short form‐ (SCNS‐SF34).^13^ The instrument consists of 5 subscales, Psychological (10 items), Health System and Information (11 items), Physical and Daily Living (5 items), Patient Care and Support (5 items), and Sexual (3 items) care needs. For this study, we added an extra dimension that we labeled as “Additional needs” (5 items) from the long version of the SCNS^14^ and refers to financial and practical difficulties. Thus, we used 39 items to measure the patients' level of need for help over 2 weeks preceding the interview. Details on the adaptation of the instrument to the Spanish version can be found elsewhere.^12^ The instrument employs a 5‐point Likert response scale to rate the intensity of each need, that is, 1 = Not applicable, 2 = Satisfied, 3 = Low need, 4 = Moderate need, and 5 = High need. A total score for each dimension was computed using standardized scores, which ranged from 0 to 100. Higher scores reflected higher supportive care needs.^15^ Cronbach alphas for the subscales at baseline ranged from .69 to .95.

*Anxiety* symptoms were measured at baseline with the short form of the Spielberg State and Trait Anxiety Inventory.^16^ This version employs 6 items from the state subscale of the long original version. We used the equivalent 6 items from the Spanish version of the instrument.^17^ A total score is obtained summing all the items after the negatives items have been properly transformed. Higher scores indicate higher levels of anxiety (1 = *not at all*, 4 = *very much*). Cronbach alpha for the scale was.81.

*Depressive* symptoms were measured at baseline with the Spanish version for Mexico of the Patient Health Questionnaire (PHQ‐9).^18^ The 9‐items instrument assesses depression severity experienced by patients in the last 2 weeks. A total score is obtained summing all the items. Higher scores indicate higher levels of depression (0 = *not at all*, 3 = *nearly every day*). Cronbach alphas for the scale was .82.

*Physical symptoms* of the disease experienced by the patients were measured after the surgery with the European Organization for Research and Treatment of Cancer: Breast Cancer Specific Quality of Life Questionnaire Module, Spanish version for Mexico (EORTC QLQ‐BR23).^19^ We used the 3 symptoms scales: systemic therapy side effects (7 items), breast symptoms (4 items), and arm symptoms (3 items). The response scale ranges from 1 = *not at all* to 4 = *very much*. Higher scores represent higher physical symptoms. Cronbach alphas for the scales ranged from .60 to.71.

At baseline, we also collected data on age, number of children, marital status, education level, work status, and life events during the last 3 months and whether patients were receiving psychological aid at the moment of the interview. Marital status was classified into with partner (married/living together/in a relationship) and without partner (single/widow/divorced); education level was classified into high (bachelor/postgraduate studies), middle (secondary/technical high school), low (without studies/primary); work status was classified into housewife or employed, and life events into yes or not. We also collected information on comorbidities, type of treatment and cancer stage.

### 2.4 Statistical analyses

We computed descriptive statistics of the sample characteristics per cancer treatment group. ANOVAs and chi‐square tests were run to compare the baseline characteristics of each group. To analyze the longitudinal course of supportive care needs, we computed linear mixed models with maximum‐likelihood estimation for each subscale separately, including group, time, and their interaction. Time is the number of days since diagnosis, and it was treated as a continuous variable because the lapse between each measuring point varied for each patient. We computed both, models with random intercept and random slope, and models with random intercept only. We used Akaike information criterion (AIC) and Bayesian information criterion (BIC) to select the best possible model. Lower values of AIC/BIC indexes were considered as indicative of a better model. According to these criteria, only the intercept was considered to be random in models for psychological, health system and information, physical and daily living, patient care and support, and sexual dimensions; whereas a random intercept and random slope were considered for additional care needs dimension. Subsequently, predictors of the supportive care needs' course were identified through univariate analyses. Those sociodemographic, physical, and psychological variables (anxiety and depressive symptoms at baseline), which were significantly related to specific supportive care needs subscale, in at least 2 measurement points were included. We recomputed linear mixed models including group, time, time × group, and the predictors for each subscale. When the interaction time × group was not significant, the model was recomputed again without the interaction. AIC/BIC decreased whenever the interaction term was removed. All patients with at least 1 observation in the measure points and with measured values of all predictors values at baseline were included in each model. Analyses were conducted with SPSS version 24. *P*‐values were 2‐sided with a significant level of 0.05.

## 3 RESULTS

### 3.1 Characteristics of the sample

Details about the flow of patients are shown in Figure 1. A total of 172 patients (66.4% response rate) agreed to participate and were assessed at baseline. Seventeen patients (9.9%) dropped out after T1. The remaining patients were classified into 3 groups based on the cancer treatment trajectory they followed: 29 in group A, 70 in group B, and 56 in group C (of whom 5 palliative patients were evaluated every 3 months after measure T2). Duration of patients' participation within the different groups differed significantly (for A, B, and C, respectively, on average 10 (SD = 3.7), 15.2 (SD = 3.5), and 16.7 (SD = 2.9) months, *P* ≤ 0.001). On average, patients were 53 years old, had middle education (60%), and had a partner (67%). Cancer stage was significantly associated to group classification, most patients with cancer stage III or IV were allocated to group C. Further details on the sample's characteristics are in supporting Table 1.

### 3.2 Course of supportive care needs

**Figure 2:**
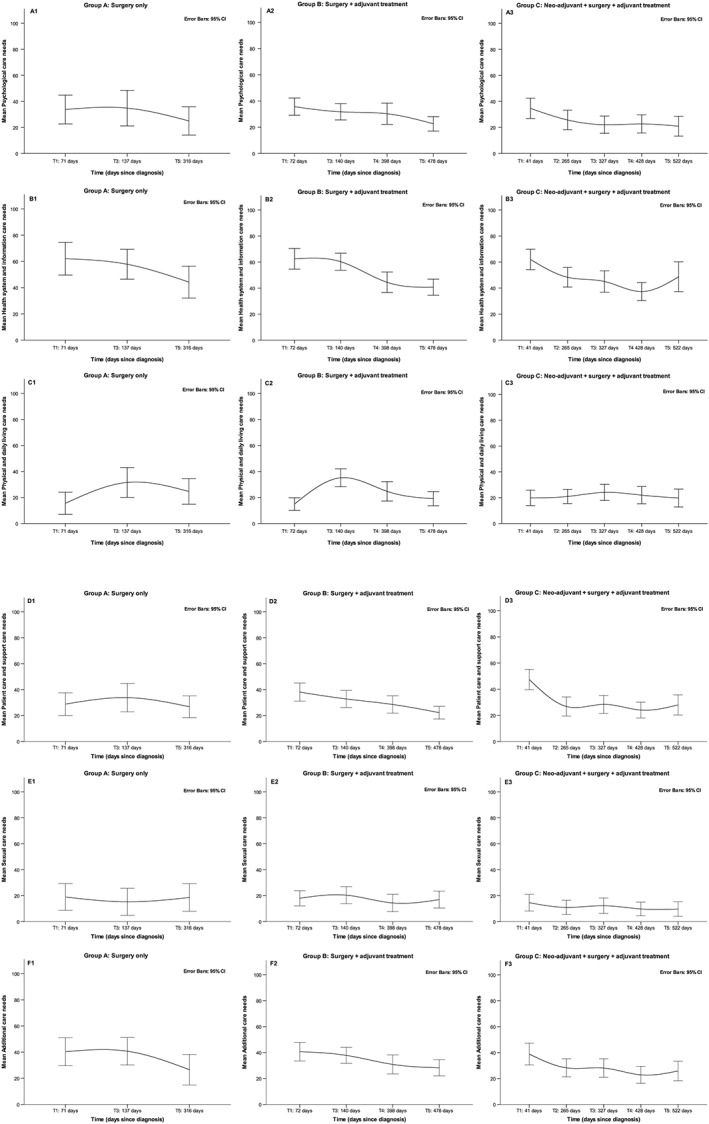
Supportive care needs course in the different medical treatment trajectory groups

For all domains, except for the Health System and Information domain in which scores ranged from moderate to low over time, scores ranged from low to no need over time (supporting table 2). The top 5 unmet needs of each domain are shown in supporting table 3. Focusing on the changes within groups, we observed a decrease in Psychological care needs over time for all groups (Figures 2A1‐2A3), but this decrease was only statistically significant for groups B and C. Health System and Information care needs also showed a significant decrease over time in all groups (Figures 2B1‐2B3). Physical and Daily Living care needs did not change significantly over time in any of the groups (Figures 2C1‐2C3). We observed a decrease in Patient Care and Support needs for all groups, but it was statistically significant only for groups B and C (Figures 2D1‐2D3). The course of Sexual care needs for groups A and B was low without significant changes over time, but there was a significant small decrease over time for group C (Figures 2E1‐2E3). The Additional care needs showed a significant decline pattern in all 3 groups (Figures 2F1‐2F3).

The interaction between days since diagnosis (time) and treatment trajectory (group) was not significant for any of the supportive care needs dimensions, suggesting that the changes on the course of supportive care needs were similar across the cancer treatment trajectories. However, we observed a significant difference in the level of Patient Care and Support dimension at T1 between the treatment trajectories of group A and group C (Table 1). On average, patients from group A at T1 started with lower levels in this dimension (Figure 2D1), compared with patients from group C (Figure 2D3, *P* = 0.02).

**Table 1:** Linear mixed models' parameter estimates for the course of supportive care needs

	Time				Group				Time × Group
		(95% CI)				(95% CI)			
Supportive care needs' domains	Estimate	Lower	Upper	*P*	Estimate	Lower	Upper	*P*	*P*
Psychological									
Group A	−0.03	−0.07	0.004	0.085	2.36	−9.83	14.55	0.704	0.953
Group B	−0.03	−0.04	−0.01	< 0.001	3.07	−6.14	12.28	0.512	0.792
Group C	−0.03	−0.04	−0.02	< 0.001					
Health system/information									
Group A	−0.06	−0.10	−0.02	0.004	4.71	−8.62	18.04	0.487	0.368
Group B	−0.05	−0.07	−0.04	< 0.001	5.99	−4.07	16.04	0.242	0.332
Group C	−0.04	−0.06	−0.02	< 0.001					
Physical/daily living									
Group A	0.01	−0.02	0.05	0.468	0.93	−9.94	11.80	0.866	0.603
Group B	−0.01	−0.02	0.01	0.354	4.57	−3.61	12.75	0.273	0.366
Group C	0.003	−0.01	0.02	0.701					
Patient care/support									
Group A	−0.01	−0.04	0.03	0.745	−13.50	−25.03	−.197	0.022	0.074
Group B	−0.03	−0.05	−0.02	< 0.001	−5.50	−14.18	3.18	0.213	0.257
Group C	−0.04	−0.06	−0.03	< 0.001					
Sexual
Group A	−0.01	−0.04	0.03	0.776	2.84	−7.83	13.51	0.601	0.601
Group B	−0.01	−0.02	0.001	0.073	5.11	−2.92	13.15	0.211	0.742
Group C	−0.01	−0.03	−0.001	0.040					
Additional									
Group A	−0.05	−0.09	−0.01	0.013	5.55	−8.15	19.25	0.425	0.476
Group B	−0.03	−0.04	−0.01	0.001	3.12	−7.30	13.54	0.555	0.685
Group C	−0.03	−0.05	−0.02	< 0.001					

Note: Group C was the reference group. Time is the number of days since diagnosis.

### 3.3 Predictors of supportive care needs

After adjusting linear mixed models by potential predictors, results showed that, in general, patients with higher levels of depressive or anxiety symptoms after diagnosis, those who received psychological aid at T1, and those with higher systemic therapy side effects after surgery showed higher care needs in specific domains over time. Specifically, older patients indicated lower Sexual care needs over time. Also, patients with a partner and those who followed surgery (Group A) or surgery plus adjuvant treatment (group B), showed higher Sexual care needs over time. The interaction time × group was not significant for any of the dimensions. Further details about predictors are in Table 2, which shows the significance and size of the fixed effects.

**Table 2:** Predictors of changes in supportive care needs based on linear mixed models

	Estimate	Lower 95% CI	Upper 95% CI
Psychological			
Time	−0.03***	−0.04	−0.02
Group (A vs C)	3.52	−4.09	11.14
Group (B vs C)	0.50	−5.60	6.60
Life events	0.85	−5.65	7.35
Anxiety	1.39***	0.64	2.14
Depression	1.44***	0.91	1.97
Psychological aid	15.60*	3.87	27.33
Side therapy effects	0.06	−0.12	0.23
Breast symptoms	0.08	−0.06	0.22
Arm symptoms	0.03	−0.10	0.16
Health system/information			
Time	−0.05***	−0.06	−0.04
Group (A vs C)	−0.63	−10.88	9.62
Group (B vs C)	0.63	−7.26	8.51
Anxiety	0.65	−0.37	1.67
Depression	0.84*	0.14	1.53
Physical/daily living			
Time	−0.002	−0.01	0.01
Group (A vs C)	2.44	−4.37	9.26
Group (B vs C)	0.65	−4.79	6.08
Life events	3.19	−2.64	9.01
Anxiety	0.87*	0.19	1.54
Depression	0.89***	0.41	1.37
Systemic therapy side effects	0.17*	0.02	0.33
Breast symptoms	0.04	−0.08	0.17
Arm symptoms	0.08	−0.04	0.19
Patient care/support			
Time	−0.04***	−0.05	−0.03
Group (A vs C)	−7.59	−15.85	0.68
Group (B vs C)	−4.40	−10.67	1.87
Anxiety	0.45	−0.36	1.27
Depression	0.94**	0.38	1.49
Sexual			
Time	−0.01*	−0.02	−0.002
Group (A vs C)	7.44*	0.04	14.85
Group (B vs C)	7.20*	1.64	12.76
Age	−0.34*	−0.61	−0.06
Marital status	11.77***	6.35	17.18
Education (low vs high)	−9.57	−19.99	0.84
Education (middle vs high)	−2.67	−11.75	6.40
Psychological aid	10.67	−1.94	23.29
Additional			
Time	−0.04***	−0.05	−0.02
Group (A vs C)	0.66	−7.80	9.11
Group (B vs C)	0.04	−6.11	6.19
Life events	3.62	−3.34	10.57
Anxiety	0.77	−0.03	1.56
Depression	1.29***	0.75	1.83
Breast symptoms	0.12	−0.02	0.25

* *P* < .05.

** *P* < .01.

*** *P* < .001.

## 4 DISCUSSION

This study was the first to examine the course of supportive care needs among Mexican breast cancer patients looking at differences in cancer treatment and potential predictors of changes in supportive care needs. Results showed that, overall the course of supportive care needs did not differ by the cancer treatment trajectory. Supportive care needs declined over time and patients reported relatively low scores. Only the levels of Health System and Information care needs were elevated over time. Depressive symptoms after diagnosis were the most consistent predictor of change in supportive care needs over time.

In general, supportive care needs of the patients were low and decreased over time, which is in line with previous studies among Chinese, Taiwanese, and French breast cancer patients.^6^, ^7^, ^8^ This might suggest that patients' needs are met; either they receive the care they require from the health system or they manage themselves to get the help they need outside, particularly within a collectivistic culture like Mexico, where social relationships and attachment between family members is highly present. Health System and Information care needs were the highest throughout time. This is consistent with previous studies among Asian patients,^6^, ^7^ but in contrast with studies among Caucasian patients where Psychological needs were also high.^8^, ^20^ This difference might be explained by the differences in the health care systems. Within the Mexican public health system is not common that patients receive extensive information about their illness and their care, consultations are rather short, and patients are not provided with brochures or leaflets about their medical condition. Thus, our findings indicate that it would be relevant to fit these aspects within the Mexican health system and to provide clear information for all breast cancer patients independent of their education level.

The only significant difference we found among patients differing in cancer treatment was a higher Patient Care and Support need at the time of diagnosis among patients who underwent neoadjuvant treatment, surgery, and adjuvant treatment, compared with those who only underwent surgery. This result may be because these patients were in advanced disease stages. Our findings contradict previous cross‐sectional studies showing differences in the health system and information,^21^ physical and daily living,^22^ and psychological care needs^9^ by the type of treatment received. This difference in results might be explained by the fact that we analyzed the effect of the whole cancer treatment trajectory on the course of supportive care needs, and the previous studies reported the single effect of specific treatment modalities, eg, chemotherapy,^7^ on patients' care needs.

Regarding the predictors of supportive care needs over time, our findings highlight the role of depressive symptoms as the main predictor of care needs, which is consistent with previous studies.^23^ Patients with higher levels of depressive symptoms after diagnosis reported higher supportive care needs over time, except for Sexual care needs. Furthermore, time since diagnosis was a consistent predictor of supportive care needs decline. These findings add to a large body of literature, both theoretical^24^, ^25^, ^26^, ^27^ and empirical,^28^ suggesting that cancer patients are rather resilient and capable of adapting to their disease.

### 4.1 Study limitations

The results of this study should be interpreted considering some limitations. In this study, physical and psychological symptoms were assessed at 1 point in time. Physical symptoms were measured after surgery, and it might be that for patients who received neoadjuvant treatment, physical symptoms started to exhibit earlier in the disease trajectory. Also, the relatively small sample size used might have prevented us from identifying a significant interaction between time and group. A higher attrition was observed among the patients in advanced cancer stage or with lower education, which is common in longitudinal studies involving (low‐middle income) cancer patients.^29^, ^30^ Although there are studies indicating that selective attrition does not always affect the estimates of associations between variables,^31^, ^32^ we advise caution in the generalizability of our findings. Because the care protocols for Mexican cancer patients might change according to the type of cancer, our findings might not be extrapolated beyond patients with breast cancer.

To the best of our knowledge, this longitudinal study is the first evaluating supportive care needs of breast cancer patients living in a Latin American country. The design of the study provides a whole picture of the care needs fluctuations since patients were followed from after diagnosis until the first post‐treatment follow‐up visit, and patients with different treatment trajectories were included. Assessments were conducted at clinical relevant points. Future research should investigate in more detail which factors are linked to unmet Health System and Information needs in these patients, whether it is related with health literacy issues, patient‐physician communication, or insufficient information provision.^33^, ^34^, ^35^ We additionally suggest piloting different intervention strategies, ie, written or web‐based information, smartphone applications, specialized nurse consultations, to meet the information needs of Mexican breast cancer patients. Also, further longitudinal studies should be done in the Latin American region to confirm our results.

### 4.2 Clinical implications

In Mexico, where the provision of supportive care for cancer patients within the public health care system has not been systematically implemented yet, our results have some implications for the delivery of such care. Health policy makers should take into account that even though Mexican breast cancer patients showed on average low supportive care needs over time, there was a moderate need of patients for more information regarding the disease, the medical treatments, and the organization of the care services within the hospital where they are being treated. Health professionals within the Mexican public health system should ensure that their patients receive the information they need regarding their illness at each phase of the disease treatment and that this information is presented in such a way that it is clear to each patient. Furthermore, clinicians should be aware that patients with depressive symptoms may be in higher need of supportive care over the course of treatment; thus, they should prioritize supportive care services for these patients. Overall, we suggest to screen the supportive care needs of Mexican breast cancer patients.

## CONFLICT OF INTEREST

None declared.

## Supporting information

Supporting table 1. Descriptive characteristics of the sample.
Supporting table 2. Mean levels of supportive care needs at each time point.
Supporting table 3: Top 5 unmet care needs per dimension based on the response rate to the low‐high need answer categories.

## References

[1] American Cancer Society . Palliative or supportive care. https://www.cancer.org/treatment/treatments-and-side-effects/palliative-care.html. Accessed October 4, 2017.

[2] Ervik M , Lam F , Ferlay J , Mery L , Soerjomataram I , Bray F . Cancer Today. Lyon, France: International Agency for Research on Cancer http://gco.iarc.fr/today/home. Accessed May 29, 2017.

[3] Cardoso F , Bese N , Distelhorst SR , et al. Supportive care during treatment for breast cancer: resource allocations in low‐ and middle‐income countries. A Breast Health Global Initiative 2013 consensus statement. The Breast. 2013;22(5):593‐605. 24001709 10.1016/j.breast.2013.07.050 PMC7442957

[4] Chávarri‐Guerra Y , Villarreal‐Garza C , Liedke PE , et al. Breast cancer in Mexico: a growing challenge to health and the health system. Lancet Oncol. 2012;13:e335–e343. 22846838 10.1016/S1470-2045(12)70246-2

[5] Instituto Nacional de Estadística y Geografía . Principales causas de mortalidad por residencia habitual, grupos de edad y sexo del fallecido. INEGI. http://www3.inegi.org.mx/sistemas/temas/default.aspx?s=est&c=17484. Accessed February 25, 2016.

[6] Liao M‐N , Chen S‐C , Chen S‐C , et al. Changes and predictors of unmet supportive care needs in Taiwanese women with newly diagnosed breast cancer. Oncol Nurs Forum. 2012;39:444. 10.1188/12.ONF.E380-E389 22940517

[7] Lam WWT , Tsang J , Yeo W , et al. The evolution of supportive care needs trajectories in women with advanced breast cancer during the 12 months following diagnosis. Support Care Cancer. 2014;22(3):635‐644. 24158684 10.1007/s00520-013-2018-x

[8] Brédart A , Merdy O , Sigal‐Zafrani B , Fiszer C , Dolbeault S , Hardouin J‐B . Identifying trajectory clusters in breast cancer survivors' supportive care needs, psychosocial difficulties, and resources from the completion of primary treatment to 8 months later. Support Care Cancer. 2016;24(1):357‐366. 26076962 10.1007/s00520-015-2799-1

[9] Lam WWT , Au AHY , Wong JHF , et al. Unmet supportive care needs: a cross‐cultural comparison between Hong Kong Chinese and German Caucasian women with breast cancer. Breast Cancer Res Treat. 2011;130(2):531‐541. 21617919 10.1007/s10549-011-1592-1

[10] Fiszer C , Dolbeault S , Sultan S , Bredart A . Prevalence, intensity, and predictors of the supportive care needs of women diagnosed with breast cancer: a systematic review. Psychooncology. 2014;23(4):361‐374. 24677334 10.1002/pon.3432

[11] National Comprehensive Cancer Network. NCCN Clinical Practice Guidelines in Oncology. Breast Cancer (version 2.2017). https://www.nccn.org/professionals/physician_gls/f_guidelines.asp. Accessed October 9, 2017. 10.6004/jnccn.2017.0146 PMC5865602 28874599

[12] Pérez‐Fortis A , Fleer J , Sánchez‐Sosa JJ , et al. Prevalence and factors associated with supportive care needs among newly diagnosed Mexican breast cancer patients. Support Care Cancer. 2017;25(10):3273‐3280. 28516220 10.1007/s00520-017-3741-5 PMC5577048

[13] Boyes A , Girgis A , Lecathelinais C . Brief assessment of adult cancer patients' perceived needs: development and validation of the 34‐item Supportive Care Needs Survey (SCNS‐SF34). J Eval Clin Pract. 2009;15(4):602‐606. 19522727 10.1111/j.1365-2753.2008.01057.x

[14] Bonevski B , Sanson‐Fisher R , Girgis A , Burton L , Cook P , Boyes A . Evaluation of an instrument to assess the needs of patients with cancer. Cancer. 2000;88(1):217‐225. 10618626 10.1002/(sici)1097-0142(20000101)88:1<217::aid-cncr29>3.0.co;2-y

[15] McElduff P , Boyes A , Zucca A , Girgis A . The Supportive Care Needs Survey: A Guide to Administration, Scoring and Analysis. Newcastle: Centre for Health Research and Psychooncology; 2004.

[16] Marteau TM , Bekker H . The development of a six‐item short‐form of the state scale of the Spielberger State—Trait Anxiety Inventory (STAI). Br J Clin Psychol. 1992;31(3):301‐306. 1393159 10.1111/j.2044-8260.1992.tb00997.x

[17] Spielberger CD , Díaz‐Guerrero R . IDARE. Inventario de Ansiedad Rasgo‐Estado. Manual e Instructivo. México: El Manual Moderno; 1975.

[18] Kroenke K , Spitzer RL , Williams JB . The Phq‐9. J Gen Intern Med. 2001;16(9):606‐613. 11556941 10.1046/j.1525-1497.2001.016009606.x PMC1495268

[19] Sprangers MA , Groenvold M , Arraras JI , et al. The European Organization for Research and Treatment of Cancer breast cancer‐specific quality‐of‐life questionnaire module: first results from a three‐country field study. J Clin Oncol. 1996;14(10):2756‐2768. 8874337 10.1200/JCO.1996.14.10.2756

[20] Schmid‐Büchi S , Halfens RJG , Müller M , Dassen T , van den Borne B . Factors associated with supportive care needs of patients under treatment for breast cancer. Eur J Oncol Nurs. 2013;17(1):22‐29. 22449715 10.1016/j.ejon.2012.02.003

[21] Au A , Lam W , Tsang J , et al. Supportive care needs in Hong Kong Chinese women confronting advanced breast cancer. Psychooncology. 2013;22(5):1144‐1151. 22715115 10.1002/pon.3119

[22] Hwang SY , Park B‐W . The perceived care needs of breast cancer patients in Korea. Yonsei Med J. 2006;47(4):524‐533. 16941743 10.3349/ymj.2006.47.4.524 PMC2687734

[23] Park BW , Hwang SY . Unmet needs of breast cancer patients relative to survival duration. Yonsei Med J. 2012;53(1):118‐125. 22187241 10.3349/ymj.2012.53.1.118 PMC3250332

[24] Helgeson VS , Zajdel M . Adjusting to chronic health conditions. Annu Rev Psychol. 2017;68(1):545‐571. 28051935 10.1146/annurev-psych-010416-044014

[25] Taylor S . Adjustment to threatening events—a theory of cognitive adaptation. Am Psychol. 1983;38(11):1161‐1173.

[26] Lazarus RS . Progress on a cognitive‐motivational‐relational theory of emotion. Am Psychol. 1991;46(8):819‐834. 1928936 10.1037//0003-066x.46.8.819

[27] Sprangers MA , Schwartz CE . Integrating response shift into health‐related quality of life research: a theoretical model. Soc Sci Med 1982. 1999;48:1507‐1515. 10.1016/s0277-9536(99)00045-3 10400253

[28] Brandão T , Schulz MS , Matos PM . Psychological adjustment after breast cancer: a systematic review of longitudinal studies. Psychooncology. 2017;26(7):917‐926. 27440317 10.1002/pon.4230

[29] Nicholson LM , Schwirian PM , Klein EG , et al. Recruitment and retention strategies in longitudinal clinical studies with low‐income populations. Contemp Clin Trials. 2011;32(3):353‐362. 21276876 10.1016/j.cct.2011.01.007 PMC3070062

[30] Perez‐Cruz PE , Shamieh O , Paiva CE , et al. Factors associated with attrition in a multicenter longitudinal observational study of patients with advanced cancer. J Pain Symptom Manage. 2018;55(3):938‐945. 29155290 10.1016/j.jpainsymman.2017.11.009 PMC5834396

[31] Gustavson K , von Soest T , Karevold E , Røysamb E . Attrition and generalizability in longitudinal studies: findings from a 15‐year population‐based study and a Monte Carlo simulation study. BMC Public Health. 2012;12(1):918. 23107281 10.1186/1471-2458-12-918 PMC3503744

[32] Lacey RJ , Jordan KP , Croft PR . Does attrition during follow‐up of a population cohort study inevitably lead to biased estimates of health status? PLoS ONE. 2013;8(12):e83948. 24386313 10.1371/journal.pone.0083948 PMC3875525

[33] Halbach SM , Ernstmann N , Kowalski C , et al. Unmet information needs and limited health literacy in newly diagnosed breast cancer patients over the course of cancer treatment. Patient Educ Couns. 2016;99(9):1511‐1518. 27378079 10.1016/j.pec.2016.06.028

[34] Faller H , Koch U , Brähler E , et al. Satisfaction with information and unmet information needs in men and women with cancer. J Cancer Surviv Res Pract. 2016;10(1):62‐70. 10.1007/s11764-015-0451-1 25956402

[35] Lie HC . Mind the gap: (unmet) information needs in cancer care. Patient Educ Couns. 2017;100(3):381‐382. 28288750 10.1016/j.pec.2017.02.013

