# Supplementary material for: Course and predictors of supportive care needs among Mexican breast cancer patients: A longitudinal study
Source: Psychooncology. 2018 Jun 19;27(9):2132–40. doi: 10.1002/pon.4778 (PMC6175400; doi:10.1002/pon.4778)
Supplement: Supplementary file 1 — Supporting table 1. Descriptive characteristics of the sample. Supporting table 2. Mean levels of supportive care needs at each time point. Supporting table 3: Top 5 unmet care needs per dimension based on the response rate to the low‐high need answer categories. [file PON-27-2132-s001.docx]

Supporting table 1. Descriptive characteristics of the sample.

| Characteristics | Group A  (n = 29) | Group B  (n = 70) | Group C  (n = 56) | Total  (n = 172) | *p*-value |
| --- | --- | --- | --- | --- | --- |
| Age (years, mean + SD) | 54.4 + 9.8 | 53.3 + 10.6 | 50.9 + 11.1 | 53.0 + 10.6 | N. S. |
| Number of children (mean + SD) | 2 + 2 | 3 + 1 | 3 + 2 | 3 + 2 | N. S. |
| Marital status |  |  |  |  | N. S. |
| With partner | 19 (65.5) | 46 (65.7) | 39 (69.6) | 116 (67.4) |  |
| Without partner | 10 (34.5) | 24 (34.3) | 17 (30.4) | 56 (32.6) |  |
| Education |  |  |  |  | N. S. |
| Low | 9 (31.0) | 18 (25.7) | 18 (32.1) | 53 (30.8) |  |
| Middle | 19 (65.5) | 45 (64.3) | 31 (55.4) | 103 (59.9) |  |
| High | 1 (3.4) | 7 (10.0) | 7 (12.5) | 16 (9.3) |  |
| Work status |  |  |  |  | N. S. |
| Employed | 8 (27.6) | 26 (37.7) | 20 (35.7) | 60 (35.1) |  |
| Housewife / retired | 21 (72.4) | 43 (62.3) | 36 (64.3) | 111 (64.9) |  |
| Life events |  |  |  |  | N. S. |
| Yes | 8 (27.6) | 22 (31.4) | 8 (14.3) | 40 (23.3) |  |
| No | 21 (72.4) | 48 (68.6) | 48 (85.7) | 132 (76.7) |  |
| Comorbidities |  |  |  |  | N. S. |
| Yes | 19 (65.5) | 39 (55.7) | 30 (53.6) | 99 (57.6) |  |
| No | 10 (34.5) | 31 (44.3) | 26 (46.4) | 73 (42.4) |  |
| Cancer stage |  |  |  |  | <0.001 |
| I | 5 (17.2) | 9 (12.9) | 3 (5.4) | 20 (11.6) |  |
| II | 15 (51.7) | 37 (52.9) | 5 (8.9) | 61 (35.5) |  |
| III-IV | 4 (13.8) | 12 (17.1) | 41 (73.2) | 66 (38.4) |  |
| Unknown | 5 (17.2) | 12 (17.1) | 7 (12.5) | 25 (14.5) |  |
| Physical symptoms |  |  |  |  |  |
| Side therapy effects (mean + SD) | 22.8 + 18.4 | 15.9 + 14.0 | 23.7 + 19.4 | 19.8 + 17.1 | 0.04 |
| Breast symptoms (mean + SD) | 35.1 + 19.2 | 29.6 + 21.4 | 30.5 + 23.4 | 31.0 + 21.6 | N. S. |
| Arm symptoms (mean + SD) | 40.1 + 17.2 | 38.5 + 26.1 | 35.1 + 21.7 | 37.7 + 23.1 | N. S. |
| Psychological aid |  |  |  |  | N. S. |
| Yes | 0 (0) | 3 (4.3) | 4 (7.1) | 8 (4.7) |  |
| No | 29 (100) | 67 (95.7) | 52 (92.9) | 164 (95.3) |  |
| Anxiety symptoms (mean + SD) | 14.0 + 4.2 | 15.4 + 4.2 | 13.9 + 3.7 | 14.5 + 4.1 | N. S. |
| Depressive symptoms (mean + SD) | 7.0 + 5.9 | 7.4 + 6.3 | 5.7 + 5.3 | 6.8 + 5.8 | N. S. |

Note. Unless the contrary is indicated, numbers represent frequencies and percentages. Seventeen patients were dropouts after the first measure, thus their **cancer** treatment trajectory is unknown and it was not possible to associate them to the **cancer** treatment groups. *P*-value reported here refers to the association between characteristics of the patients and the cancer treatment group.

Supporting table 2. Mean levels of supportive care needs at each time point.

| Supportive care needs dimension | T1  Mean + SD | T2  Mean + SD | T3  Mean + SD | T4  Mean + SD | T5  Mean + SD |
| --- | --- | --- | --- | --- | --- |
| Psychological |  |  |  |  |  |
| Group A | 33.79 + 29.13 |  | 34.83 + 35.99 |  | 25.00 + 26.95 |
| Group B | 35.68 + 27.59 |  | 31.75 + 26.08 | 30.28 + 29.72 | 22.62 + 21.50 |
| Group C | 34.64 + 29.10 | 25.74 + 27.56 | 22.09 + 23.05 | 22.68 + 22.16 | 20.95 + 22.92 |
| Health system and information |  |  |  |  |  |
| Group A | 62.07 + 32.92 |  | 57.84 + 29.91 |  | 44.23 + 30.11 |
| Group B | 62.47 + 33.38 |  | 60.23 + 27.35 | 44.44 + 28.95 | 40.69 + 24.25 |
| Group C | 61.97 + 29.19 | 48.32 + 27.69 | 45.04 + 28.53 | 37.31 + 21.89 | 48.71 + 34.35 |
| Physical and daily living |  |  |  |  |  |
| Group A | 15.52 + 22.53 |  | 31.55 + 30.12 |  | 24.62 + 24.37 |
| Group B | 14.93 + 20.58 |  | 35.14 + 28.86 | 24.72 + 27.39 | 19.10 + 21.38 |
| Group C | 19.82 + 22.28 | 20.93 + 20.14 | 24.08 + 21.79 | 21.95 + 21.06 | 19.73 + 20.82 |
| Patient care and support |  |  |  |  |  |
| Group A | 28.79 + 22.94 |  | 33.79 + 28.87 |  | 26.73 + 20.78 |
| Group B | 38.07 + 29.42 |  | 32.79 + 27.70 | 28.52 + 24.49 | 22.21 + 18.99 |
| Group C | 47.41 + 29.09 | 26.76 + 26.87 | 28.37 + 23.94 | 24.02 + 19.24 | 27.97 + 23.11 |
| Sexual |  |  |  |  |  |
| Group A | 18.97 + 27.09 |  | 15.23 + 27.47 |  | 18.59 + 26.28 |
| Group B | 17.98 + 24.72 |  | 20.36 + 27.75 | 14.35 + 24.52 | 16.94 + 25.41 |
| Group C | 14.58 + 24.01 | 10.96 + 20.01 | 12.25 + 20.77 | 9.76 + 16.76 | 9.69 + 16.84 |
| Additional |  |  |  |  |  |
| Group A | 40.35 + 28.12 |  | 40.69 + 27.61 |  | 26.54 + 28.94 |
| Group B | 40.64 + 30.10 |  | 37.79 + 25.73 | 30.83 + 26.95 | 28.20 + 24.21 |
| Group C | 38.84 + 31.41 | 28.24 + 25.52 | 28.06 + 24.98 | 22.81 + 20.83 | 25.81 + 22.53 |

Supporting table 3: Top five unmet care needs per dimension based on the response rate to the low-high need answer categories.

|  | **T1 (n = 173)** | | | | | | | **T2 (n = 54)** | | | | | | | **T3 (n = 149)** | | | | | **T4 (n = 94)** | | | | | | | **T5 (n = 125)** | | | | | | |
| --- | --- | --- | --- | --- | --- | --- | --- | --- | --- | --- | --- | --- | --- | --- | --- | --- | --- | --- | --- | --- | --- | --- | --- | --- | --- | --- | --- | --- | --- | --- | --- | --- | --- |
| **Unmet care need by dimension** | **n** | **%** | | | **Rank** | | | **n** | **%** | | | **Rank** | | | **n** | **%** | **Rank** | | | **n** | **%** | | | **Rank** | | | **n** | **%** | | | **Rank** | | |
| **Psychological** |  | | | | | | |  | | | | | | |  | | | | |  | | | | | | |  | | | | | | |
| Worry that the results of treatment are beyond your control | 95 | | 55 | | | 1 | | 16 | | 30 | | | 5 | | 69 | 46 | | 1 | | 35 | | 37 | | | 4 | | 42 | | 34 | | | 5 | |
| Fears about the cancer spreading | 93 | | 54 | | | 2 | | 24 | | 44 | | | 2 | | 64 | 43 | | 4 | | 42 | | 45 | | | 3 | | 58 | | 46 | | | 1 | |
| Concerns about the worries of those close to you | 89 | | 51 | | | 3 | | 25 | | 46 | | | 1 | | 68 | 46 | | 3 | | 48 | | 51 | | | 1 | | 55 | | 44 | | | 2 | |
| Uncertainty about the future | 86 | | 50 | | | 4 | | 21 | | 39 | | | 3 | | 68 | 46 | | 2 | | 42 | | 45 | | | 2 | | 47 | | 38 | | | 3 | |
| Learning to feel in control of your situation | 81 | | 47 | | | 5 | | 18 | | 33 | | | 4 | | 57 | 38 | | 5 | | 34 | | 36 | | | 5 | | 43 | | 34 | | | 4 | |
| **Health system/information** |  | | | | | | |  | | | | | | |  | | | | |  | | | | | | |  | | | | | | |
| Being informed about things you one can do to help oneself to get well | 134 | | | 78 | | 1 | | 36 | | | 67 | | | 1 | 106 | 71 | | | 1 | 52 | | | 55 | | | 1 | 74 | | | 59 | | | 1 |
| Being informed whether cancer is under control or diminishing | 125 | | | 72 | | 2 | | 31 | | | 57 | | | 3 | 98 | 66 | | | 3 | 47 | | | 50 | | | 3 | 68 | | | 54 | | | 4 |
| Having access to professional counselling if the patient, family, or friends need it | 124 | | | 72 | | 3 | |  | | |  | | | - |  |  | | | - |  | | |  | | | - | 64 | | | 51 | | | 5 |
| Having one member of hospital staff to talk about all aspects of one’s condition, treatment, and follow-up | 124 | | | 72 | | 4 | |  | | |  | | | - | 87 | 58 | | | 5 |  | | |  | | | - |  | | |  | | | - |
| Receiving explanations of tests when desired | 123 | | | 71 | | 5 | | 34 | | | 63 | | | 2 | 101 | 68 | | | 2 | 49 | | | 52 | | | 2 | 69 | | | 55 | | | 3 |
| Receiving written information about the important aspects of one’s care |  | | |  | | - | | 31 | | | 57 | | | 4 |  |  | | | - |  | | |  | | | - |  | | |  | | | - |
| Being adequately informed about the benefits and side-effects of treatments before choosing to have them |  | | |  | | - | | 30 | | | 56 | | | 5 | 91 | 61 | | | 4 | 47 | | | 50 | | | 4 | 70 | | | 56 | | | 2 |
| Receiving information about aspects of managing one’s illness and side-effects at home |  | | |  | | - | | 30 | | | 56 | | | 5 | 87 | 58 | | | 5 |  | | |  | | | - |  | | |  | | | - |
| Being informed about test results as soon as feasible |  | | |  | | - | |  | | |  | | | - |  |  | | | - | 43 | | | 46 | | | 5 |  | | |  | | | - |
| **Physical/daily living** |  | | | | | | |  | | | | | | |  | | | | |  | | | | | | |  | | | | | | |
| Not being able to do the things you used  to do | 47 | | | 27 | | | 1 | 25 | | | 46 | | | 1 | 73 | 49 | | | 1 | 37 | | | 39 | | | 1 | 56 | | | 49 | | | 1 |
| Feeling unwell a lot of the time | 32 | | | 19 | | | 2 | 13 | | | 24 | | | 2 | 44 | 30 | | | 5 | 21 | | | 22 | | | 5 | 24 | | | 19 | | | 4 |
| Work around the home | 30 | | | 17 | | | 3 | 11 | | | 20 | | | 3 | 54 | 36 | | | 3 | 25 | | | 27 | | | 3 | 37 | | | 30 | | | 2 |
| Lack of energy/tiredness | 29 | | | 17 | | | 4 | 11 | | | 20 | | | 4 | 55 | 37 | | | 2 | 28 | | | 30 | | | 2 | 29 | | | 23 | | | 3 |
| Pain | 27 | | | 16 | | | 5 | 5 | | | 9 | | | 5 | 46 | 31 | | | 4 | 23 | | | 25 | | | 4 | 12 | | | 10 | | | 5 |
| **Patient care/support** |  | | | | | | |  | | | | | | |  | | | | |  | | | | | | |  | | | | | | |
| Hospital staff acknowledging, showing sensitivity to feelings and emotional needs | 79 | | | 46 | | | 1 | 16 | | | 30 | | | 1 | 51 | 34 | | | 3 | 32 | | | 34 | | | 3 | 41 | | | 33 | | | 2 |
| Reassurance by medical staff that the way you feel is normal | 79 | | | 46 | | | 2 | 11 | | | 20 | | | 4 | 59 | 40 | | | 1 | 34 | | | 36 | | | 1 | 46 | | | 37 | | | 1 |
| Hospital staff attending promptly physical needs | 77 | | | 45 | | | 3 | 13 | | | 24 | | | 2 | 54 | 36 | | | 2 | 32 | | | 34 | | | 2 | 39 | | | 31 | | | 3 |
| More choice about which cancer specialists you see | 70 | | | 41 | | | 4 | 13 | | | 24 | | | 3 | 38 | 26 | | | 4 | 17 | | | 18 | | | 4 | 24 | | | 19 | | | 4 |
| More choice about which hospital you attend | 50 | | | 29 | | | 5 | 8 | | | 15 | | | 5 | 28 | 19 | | | 5 | 9 | | | 10 | | | 5 | 10 | | | 8 | | | 5 |
| **Sexual** |  | | | | | | |  | | | | | | |  | | | | |  | | | | | | |  | | | | | | |
| Being given information on sexual relationships | 58 | | | 34 | | | 1 | 9 | | | 18 | | | 1 | 48 | 32 | | | 1 | 19 | | | 20 | | | 1 | 35 | | | 28 | | | 1 |
| Changes in sexual feelings | 29 | | | 17 | | | 2 | 7 | | | 13 | | | 2 | 23 | 15 | | | 2 | 13 | | | 14 | | | 2 | 21 | | | 17 | | | 2 |
| Changes in sexual relationships | 21 | | | 12 | | | 3 | 7 | | | 13 | | | 3 | 18 | 12 | | | 3 | 9 | | | 10 | | | 3 | 17 | | | 14 | | | 3 |
| **Additional** |  | | | | | | |  | | | | | | |  | | | | |  | | | | | | |  | | | | | | |
| Opportunity to talk to someone who understands and has been through a similar experience | 94 | | | 54 | | | 1 | 16 | | | 30 | | | 3 | 64 | 43 | | | 3 | 28 | | | 30 | | | 3 | 36 | | | 29 | | | 3 |
| Concerns about the financial situation | 83 | | | 48 | | | 2 | 23 | | | 43 | | | 1 | 78 | 52 | | | 1 | 42 | | | 45 | | | 1 | 60 | | | 48 | | | 1 |
| Family or friends to be allowed with the patient in hospital whenever the patient wants | 72 | | | 42 | | | 3 | 15 | | | 28 | | | 5 | 41 | 28 | | | 5 | 17 | | | 18 | | | 5 | 28 | | | 22 | | | 5 |
| Concerns about getting to and from the hospital | 69 | | | 40 | | | 4 | 15 | | | 28 | | | 4 | 64 | 43 | | | 2 | 30 | | | 32 | | | 2 | 39 | | | 31 | | | 2 |
| Changes in other people’s attitudes and behavior towards the patient | 54 | | | 31 | | | 5 | 18 | | | 33 | | | 2 | 43 | 29 | | | 4 | 28 | | | 30 | | | 4 | 32 | | | 26 | | | 4 |
